# Differential response to hepatic differentiation stimuli of amniotic epithelial cells isolated from four regions of the amniotic membrane

**DOI:** 10.1111/jcmm.14928

**Published:** 2020-03-06

**Authors:** Francesca Passaretta, Domenico Bosco, Lucia Centurione, Maria Antonietta Centurione, Fabio Marongiu, Roberta Di Pietro

**Affiliations:** ^1^ Department of Medicine and Ageing Sciences Gabriele D'Annunzio University of Chieti‐Pescara Chieti Italy; ^2^ StemTeCh Group Chieti Italy; ^3^ Institute of Molecular Genetics National Research Council‐Pavia Section of Chieti Chieti Italy; ^4^ Department of Biomedical Sciences Unit of Experimental Medicine University of Cagliari Cagliari Italy

**Keywords:** amniotic epithelial cells, amniotic membrane, cell transplantation, hepatocytes, human term placenta, stem cell differentiation

## Abstract

Human Amniotic Epithelial Cells (hAEC) isolated from term placenta are a promising source for regenerative medicine. However, it has long been debated whether the hAEC population consists of heterogeneous or homogeneous cells. In a previous study, we investigated the characteristics of hAEC isolated from four different regions of the amniotic membrane finding significant heterogeneity. The aim of this study was to evaluate the hepatic differentiation capability of hAEC isolated from these four regions. Human term placentae were collected after caesarean section and hAEC were isolated from four regions of the amniotic membrane (R1‐R4, according to their relative distance from the umbilical cord) and treated in hepatic differentiation conditions for 14 days. hAEC‐derived hepatocyte‐like cells showed marked differences in the expression of hepatic markers: R4 showed higher levels of Albumin and Hepatocyte Nuclear Factor (HNF) 4α whereas R1 expressed higher Cytochrome P450 enzymes, both at the gene and protein level. These preliminary results suggest that hAEC isolated from R1 and R4 of the amniotic membrane are more prone to hepatic differentiation. Therefore, the use of hAEC from a specific region of the amniotic membrane should be taken into consideration as it could have an impact on the outcome of therapeutic applications.

## 1 INTRODUCTION

Liver transplantation is still regarded as the only therapeutic option for end‐stage liver disease and for metabolic liver‐based disorders.^1^ Due to the increasing demand and the limited availability of useful organs, transplantation of isolated hepatocytes has been proposed as an alternative to whole organ transplant, particularly for the correction of metabolic defects where there is one single missing function in an otherwise proficient liver.^1^, ^2^ In an effort to find a reliable and abundant source of hepatocytes, several stem cell types have been investigated for their ability to differentiate into liver cells.

Among stem cells, human amniotic epithelial cells (hAEC) derived from term placenta represent a promising resource for regenerative medicine as they have the ability to differentiate into cells from all three germ layers, they are not tumorigenic and they possess peculiar anti‐inflammatory and immunomodulatory properties.^3^ Most importantly, we reported that hAEC can differentiate in vitro into hepatocyte‐like cells.^4^ Moreover, when transplanted into the liver of experimental animals, both human‐ and rat‐derived AEC were able to engraft and differentiate into mature hepatocytes, with no evidence of fusion with resident cells.^4^, ^5^

Morphological and functional heterogeneity of hAEC isolated from different regions of the human amniotic membrane (hAM) is still debated. While most reports consider the hAM in toto, very few studies have attempted to address this issue, and results are so far limited and sometimes conflicting.^6^, ^7^, ^8^, ^9^

In a recent study, we showed that hAEC isolated from different regions of the hAM have different expression of pluripotency and proliferation markers, different proliferative capability and different osteogenic potential.^10^ Moreover, expression of the early hepatic marker α‐fetoprotein was prominent in the region closest to the umbilical cord.^10^

In light of these considerations, the aim of this study was to investigate the ability of hAEC isolated from four regions of the hAM to differentiate in vitro into hepatocytes and to unveil possible differences among these regions.

## 2 MATERIALS AND METHODS

### 2.1 Isolation of hAEC

Ten human term placentas were collected from healthy women undergoing caesarean section after obtaining informed written consent according to the guidelines set by the Ethics Committee of the University of Chieti–Pescara. Placentas were dissected, and hAM was peeled off from four different concentric regions, identified regions according to their macroscopic characteristics and position relative to umbilical cord (Figure S1A): R1 = surrounding the umbilical cord; R2 = intermediate between R1 and R3; R3 = peripheral to the placental disc; R4 = reflected hAM.^10^ Human amniotic epithelial cells were isolated via trypsin/EDTA 0.05% digestion as previously described.^11^ Cell viability was assessed by Trypan Blue dye exclusion and consistently exceeded 90%.

### 2.2 Hepatic differentiation of hAEC

After isolation, hAEC were maintained for 3‐6 days in Dulbecco's Modified Eagle Medium (DMEM high glucose, Thermo Fisher Scientific) with standard supplements (Std) defined as follows: 10% FBS, 1% L‐glutamine (200 mmol/L), 1% non‐essential amino acids, 55 μmol/L 2‐mercaptoethanol, 1% sodium pyruvate (100 mmol/L) and 1% antibiotic/antimycotic (all from Thermo Fisher Scientific) and further supplemented with 10 ng/mL epidermal growth factor (Peprotech).

Hepatic differentiation was induced as previously described^4^ with some modifications (Figure S1B). Culture plates were coated with three different substrates: laminin 521, 422 (both from BioLamina) or Geltrex (Thermo Fisher Scientific). All substrates were used at a final concentration of 10 μg/mL and were allowed to polymerize (overnight at 4°C for laminins, 30 minutes at 37°C for Geltrex). Sub‐confluent hAEC monolayers were treated with 0.25% Trypsyn/EDTA solution, and isolated cells were seeded on previously coated plates at a density of ~1 × 10^5^ cells/cm^2^ in DMEM Std + 10 ng/mL EGF and cultured for 24 hours. Cells were then overlayed with 0.44 mg/mL of Geltrex and cultured for 48 hours. Medium was switched to IMDM Std + 10% FBS + 10 ng/mL EGF + 10 ng/mL bFGF (Peprotech) and cultured for 2 days, then added with 20 ng/mL HGF (Peprotech), 1 μmol/L Dex (Sigma Aldrich) 1× Insulin/Transferrin/Selenium (ITS) (Thermo Fisher Scientific) and cultured for further 5 days. For an additional week, the treatment was maintained with the exception of FGF‐2 which was replaced by 20 ng/mL oncostatin‐M (OSM) (Peprotech).

### 2.3 Additional materials and methods

Detailed description of materials and methods for transmission electron microscopy, RNA isolation, qRT‐PCR, immunofluorescence and statistical analysis is provided in supporting information.

## 3 RESULTS

### 3.1 Laminin 521 enhances hepatic differentiation of hAEC

**Figure 1:**
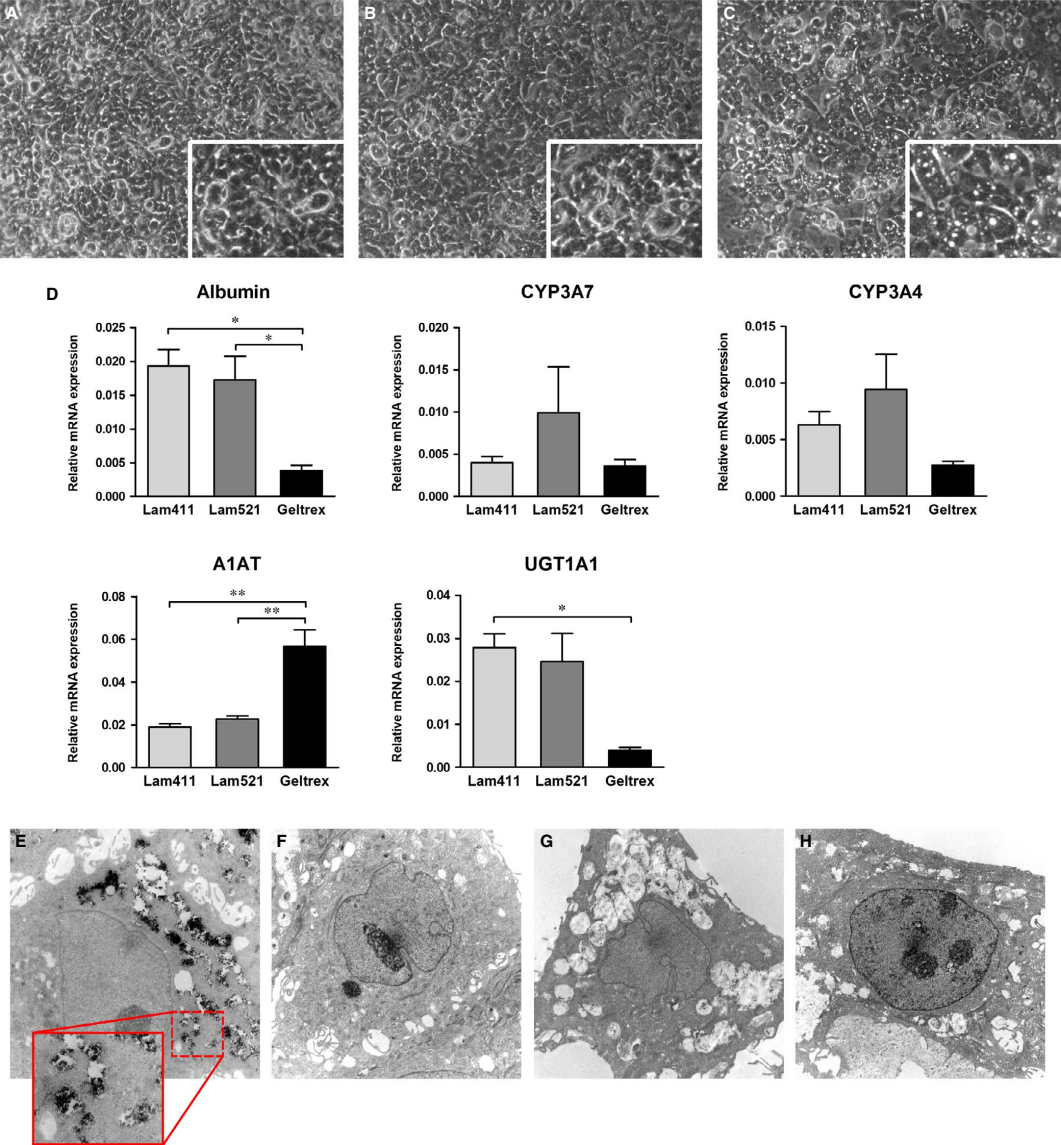
Cell morphology of hAEC‐Hep derived from a pool of all four regions and differentiated on laminin 411 (A), laminin 521 (B) and Geltrex (C) (magnification 100×; inset magnification 200×). (D) Gene expression analysis of hAEC‐Hep derived from a pool of all four regions and differentiated on three culture substrates, relative to β_2_ microglobulin (n = 3). *P < .05; **P < .01. (E‐H) Ultrastructural analysis of hAEC‐Hep derived from each single region of hAM: representative images of regions R1 to R4, respectively. Magnification 4400×. Inset shows 12 000× magnification

Previous studies on the differentiation of hAEC into hepatocytes utilized in‐house manufactured pig liver‐derived extracellular matrix (ECM) as a culture substrate.^4^ In this study, we sought to identify a suitable commercially available alternative substrate. Engelbreth‐Holm‐Swarm tumour‐derived ECM (commercially known as Matrigel or Geltrex) has long been described as a suitable substrate for the maintenance of primary human hepatocyte cultures.^12^ More recently, recombinant laminins, major components of basal membranes, have been shown to successfully maintain hepatic function in human hepatocytes in vitro.^13^ Moreover, laminin 411 and 521 were used for the differentiation and maintenance of induced pluripotent stem cells (iPS)‐derived hepatocytes.^14^, ^15^ We tested the efficacy of laminin 411, 521 and Geltrex as a culture substrate for the differentiation of hAEC into hepatocyte‐like cells. While the morphology was more similar to mature hepatocytes when cells were cultured on Geltrex (Figure 1A‐C), gene expression analysis revealed that hAEC‐derived hepatocyte‐like cells (hAEC‐Hep) had higher levels of hepatic marker genes (ie albumin, UDP glucuronosil transferase [UGT1A1]) when cultured on laminin 521 as compared to laminin 411 or Geltrex (Figure 1D).

### 3.2 hAEC from R1 and R4 are more prone to hepatic differentiation

In order to evaluate the hepatic differentiation ability of hAEC isolated from the four regions, cells were cultured on laminin 521 in the presence of inducive culture conditions for 2 weeks.

Phase contrast analysis of cell morphology showed significant differences between undifferentiated hAEC and hAEC‐Hep, although no apparent differences could be observed among cells from different regions (Figure S2). Ultrastructural analysis (Figure 1E‐H) of hAEC‐Hep monolayers showed the presence of round/polygonal epithelial cells with round central euchromatic nuclei with one or more evident nucleoli in all the 4 regions of the amniotic membrane. The cell cytoplasm of hAEC‐Hep displayed morphological characteristics commonly found in hepatocytes in all 4 regions, but mainly in R1 and R3. Such features included an evident rough endoplasmic reticulum and a number of vacuoles and dilated cisternae of smooth endoplasmic reticulum. Interestingly, electron microscopy revealed the presence of glycogen granules (dark rosettes) exclusively in the cytoplasm of hAEC‐Hep from R1 inside the dilated cisternae of smooth endoplasmic reticulum that are known to be involved in glycogen metabolism (Figure 1E, inset).

**Figure 2:**
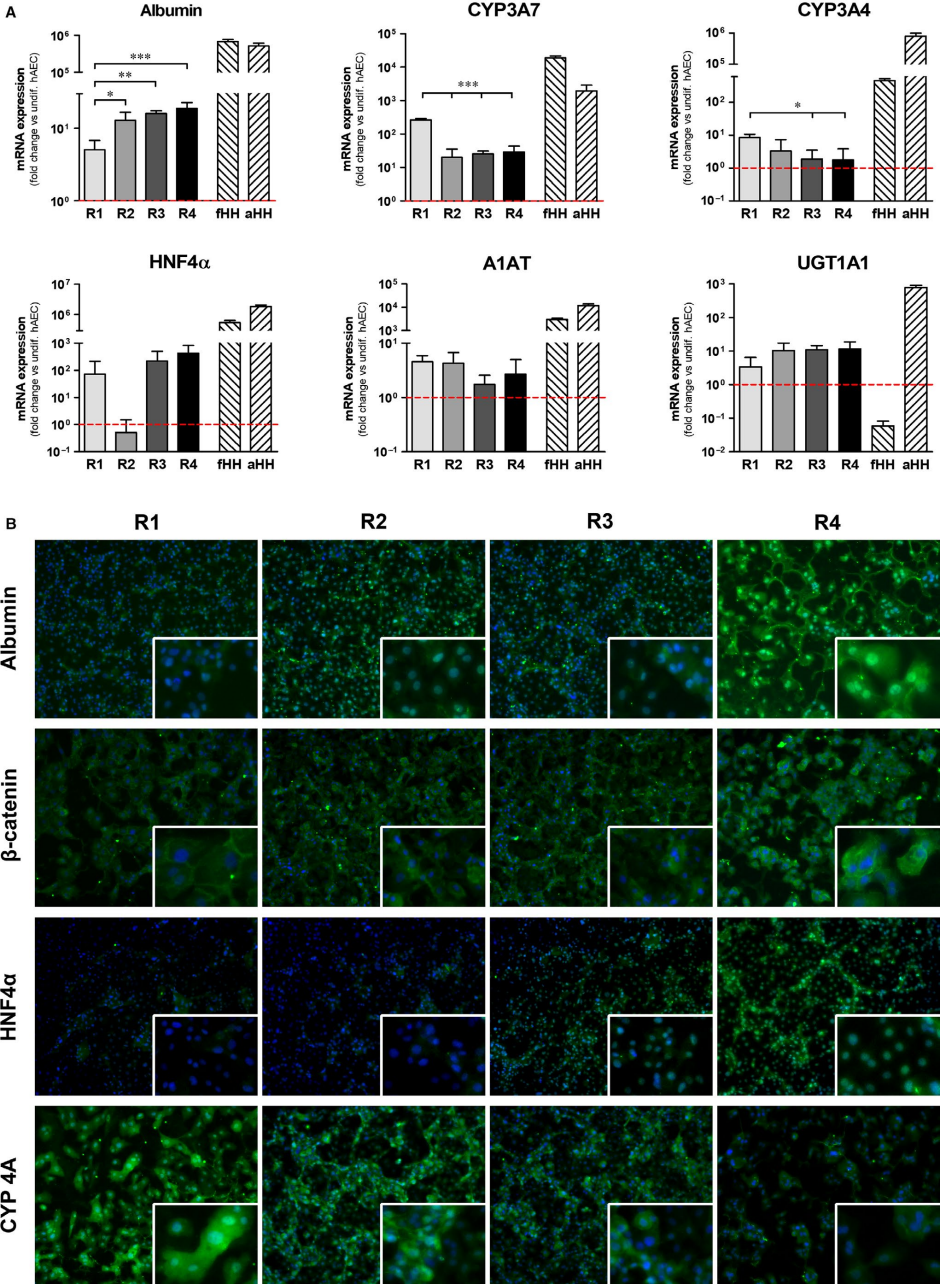
Hepatic differentiation of hAECs isolated from four regions of hAM. (A) Gene expression analysis of hepatic markers on hAEC subpopulations differentiated on laminin 521 (n = 5). Values were normalized to β2 microglobulin expression and presented as fold‐change versus undifferentiated hAEC (n = 5). fHH = foetal human hepatocytes (n = 5); aHH = adult human hepatocytes (n = 8). (*P < .05; **P < .01; ***P < .001). (B) Representative immunofluorescence images of marker proteins typical of hepatocytes on hAEC‐Hep from four regions differentiated on laminin 521 (magnification 200×; inset magnification 400×). Semi‐quantitative analysis of fluorescence intensity is available in Figure S3

Gene expression analysis revealed that hAEC‐Hep derived from R4 had significantly higher levels of albumin, and higher levels of hepatocyte nuclear factor 4 alpha (HNF4α), although this was not statistically significant (Figure 2A). On the other hand, cells from R1 expressed significantly higher levels of cytochrome P450 (CYP) 3A7 and 3A4 and higher levels of alpha‐1‐antitrypsin (A1AT) as compared to other regions (Figure 2A). A detailed comparison with gene expression levels of foetal and adult human hepatocytes is available in Table S2.

Further investigation with immunofluorescence confirmed that hAEC‐Hep from R4 had higher protein expression of albumin and HNF4α, whereas higher levels of CYP 4A were detected in cells from R1 (Figure 2B and Figure S3). Moreover, specific sub‐membrane localization of β‐catenin was prominent in hAEC‐Hep from R4 and, at lower levels, in those from R1 (Figure 2B).

## 4 DISCUSSION

In the last decade, hAEC are being considered as possible alternative source of hepatocytes for a number of clinical applications, including the correction of liver‐based metabolic diseases.^1^ Therefore, it appears important to test the hypothesis that hAEC isolated from different regions of the hAM might possess peculiar plasticity and hepatic differentiation potential.

In the current study, a previously published protocol for the differentiation of hAEC into hepatocyte‐like cells was adapted for the use with commercially available culture substrates. Laminin 521, which is a major component of basal membranes, has been shown to successfully maintain hepatic function in human hepatocytes cultures, and to promote differentiation and maintenance of iPS‐derived hepatocytes.^13^, ^15^ Here, we showed that the hepatic differentiation of hAEC cultured on laminin 521 was enhanced when compared to laminin 411 or Geltrex. Moreover, hAEC‐hep derived from R1 and R4 showed consistently higher levels of expression of hepatic marker genes and proteins, as compared to R2 and R3. Interestingly, most of the markers were expressed at higher levels in R4, including albumin, HNF4α and β‐catenin, while, conversely, all cytochrome P450 enzymes analysed were significantly higher in R1. Additionally, ultrastructural analysis revealed the presence in all the 4 regions of round euchromatic nuclei with one or more prominent nucleoli and bountiful quantities of both rough and smooth endoplasmic reticulum with dilated cisternae in the cytoplasm, all morphological characteristics suggestive of hepatocyte‐like cells. Interestingly, the presence of glycogen granules typical of functional hepatocytes was exclusively detected in hAEC‐Hep from R1.

Although preliminary, these results suggest that hAEC isolated from different regions of the hAM have heterogeneous responses to differentiative stimuli, and that cells from R1 and R4 are more susceptible to acquire phenotypic features of hepatocytes, although in a differential manner. This adds new insights into hAEC heterogeneity and highlights the need for further phenotypical characterization and functional studies to elucidate the biological significance of these differences. Most importantly, the use of hAEC from a specific region of AM should be taken into consideration towards improving the outcome of future therapeutic applications.

## CONFLICT OF INTEREST

All authors declare no conflict of interest.

## AUTHOR'S CONTRIBUTIONS

FP carried out the experiments and wrote the manuscript; LC analysed ultrastructural data; DB and MAC carried out electron microscopy procedures; FM and RDP designed the study, discussed findings and critically revised the manuscript.

## Data Availability

The data that support the findings of this study are available from the corresponding author upon reasonable request.
