# Supplementary material for: Differential response to hepatic differentiation stimuli of amniotic epithelial cells isolated from four regions of the amniotic membrane
Source: J Cell Mol Med. 2020 Mar 6;24(7):4350–5. doi: 10.1111/jcmm.14928 (PMC7171396; doi:10.1111/jcmm.14928)
Supplement: Supplementary file 1 [file JCMM-24-4350-s001.pdf]

SUPPORTING INFORMATION

Supporting Figure 1. Schematic representation of experimental protocols. (A) Isolation of hAEC from four regions (R1 to R4) of the hAM and in vitro seeding for hepatic differentiation. R1 = surrounding the umbilical cord; R2 = intermediate between R1 and R3; R3 = peripheral to the placental disc; R4 = reflected hAM. (B) Hepatic differentiation protocol.

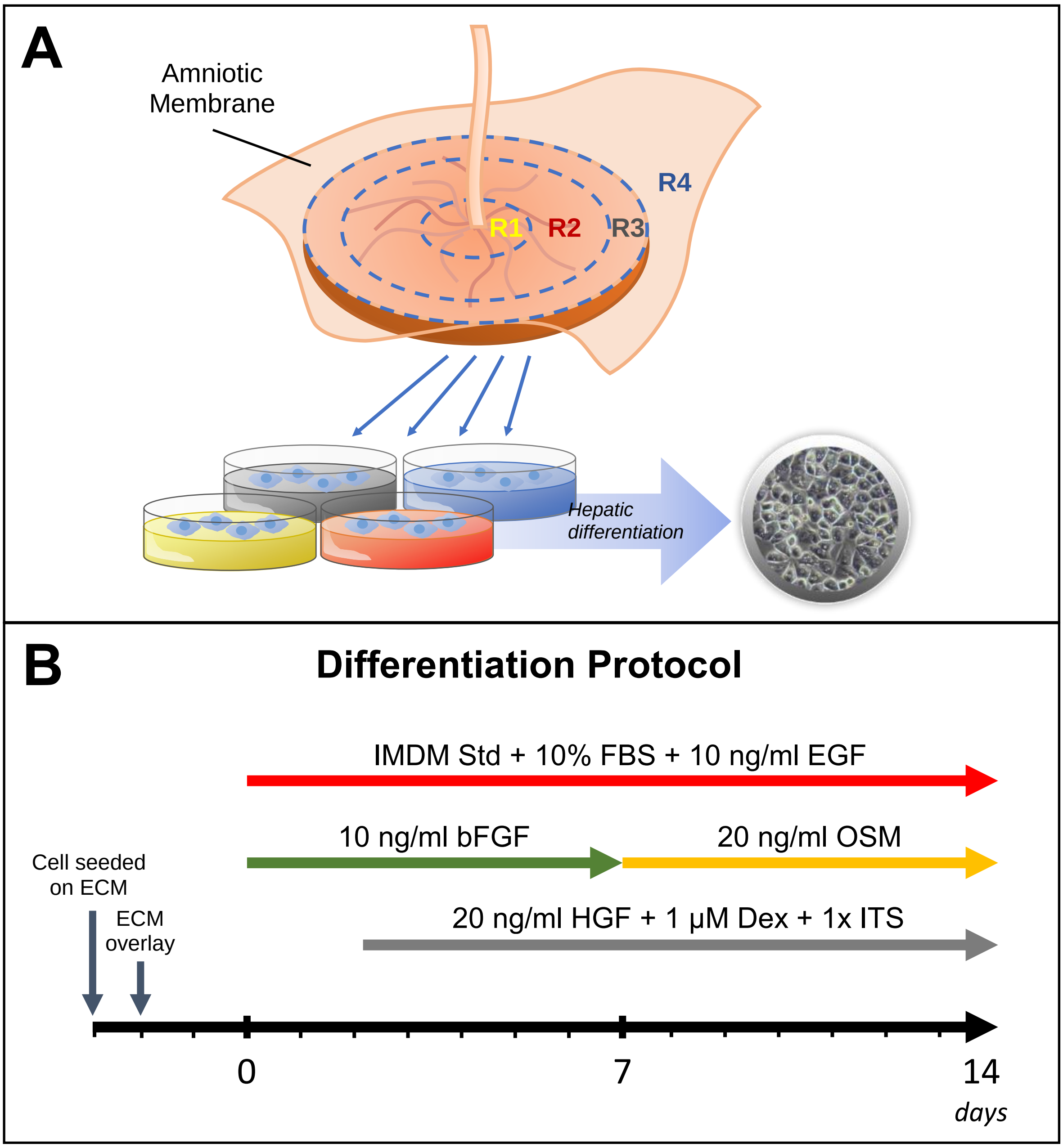

Supporting Figure 2. Cell morphology of undifferentiated hAEC and hAEC-Hep from four different regions (magnification 200×). (A) Undifferentiated hAEC seeded on laminin 521 for 48h in DMEM with standard supplements. (B-C) hAEC-Hep differentiated on laminin 521 at the end of the treatment. (B) Fields showing confluent cells. (C) Fields showing subconfluent cells.

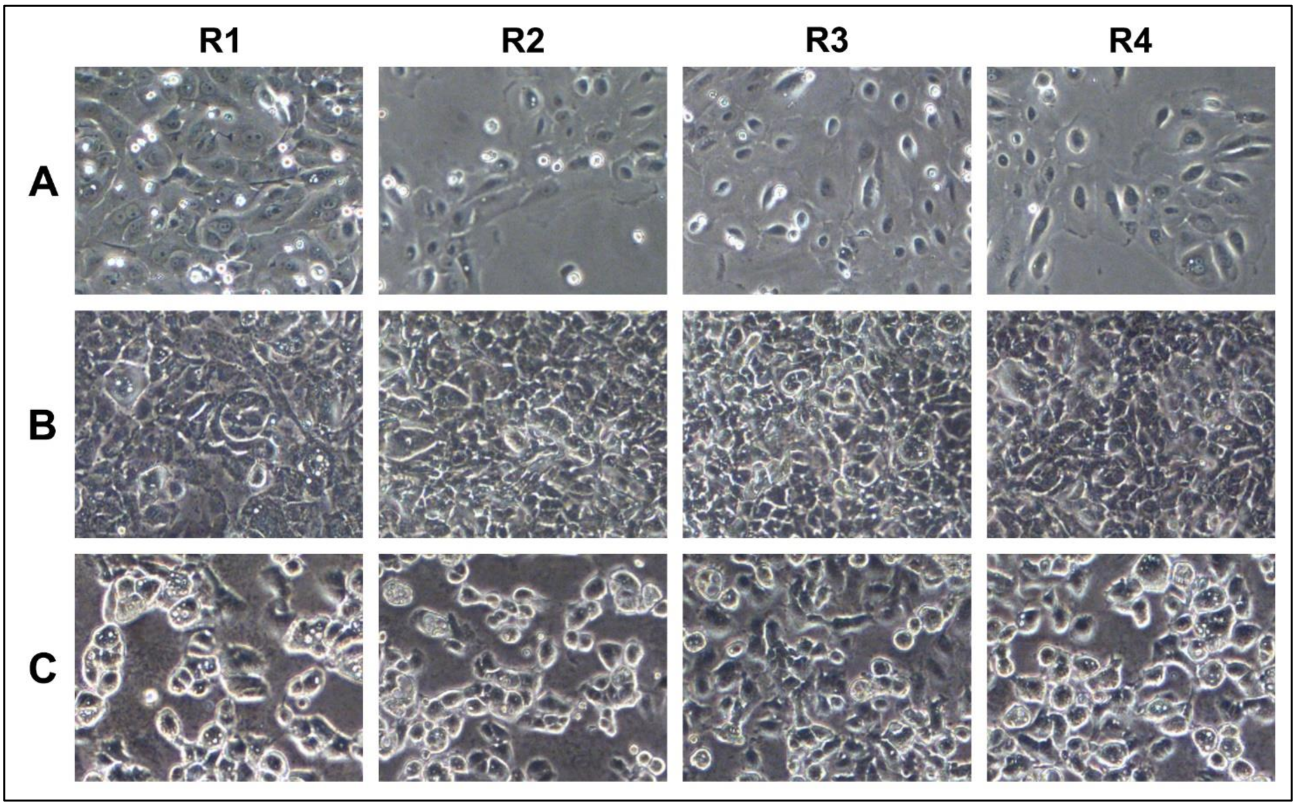

Supporting Figure 3. Analysis of immunofluorescence in hAEC-Hep differentiated on laminin 521 at the end of the treatment. For each region, a total of 8 fields per sample (200x images; n=3) were analysed and mean green fluorescence intensity was measured.

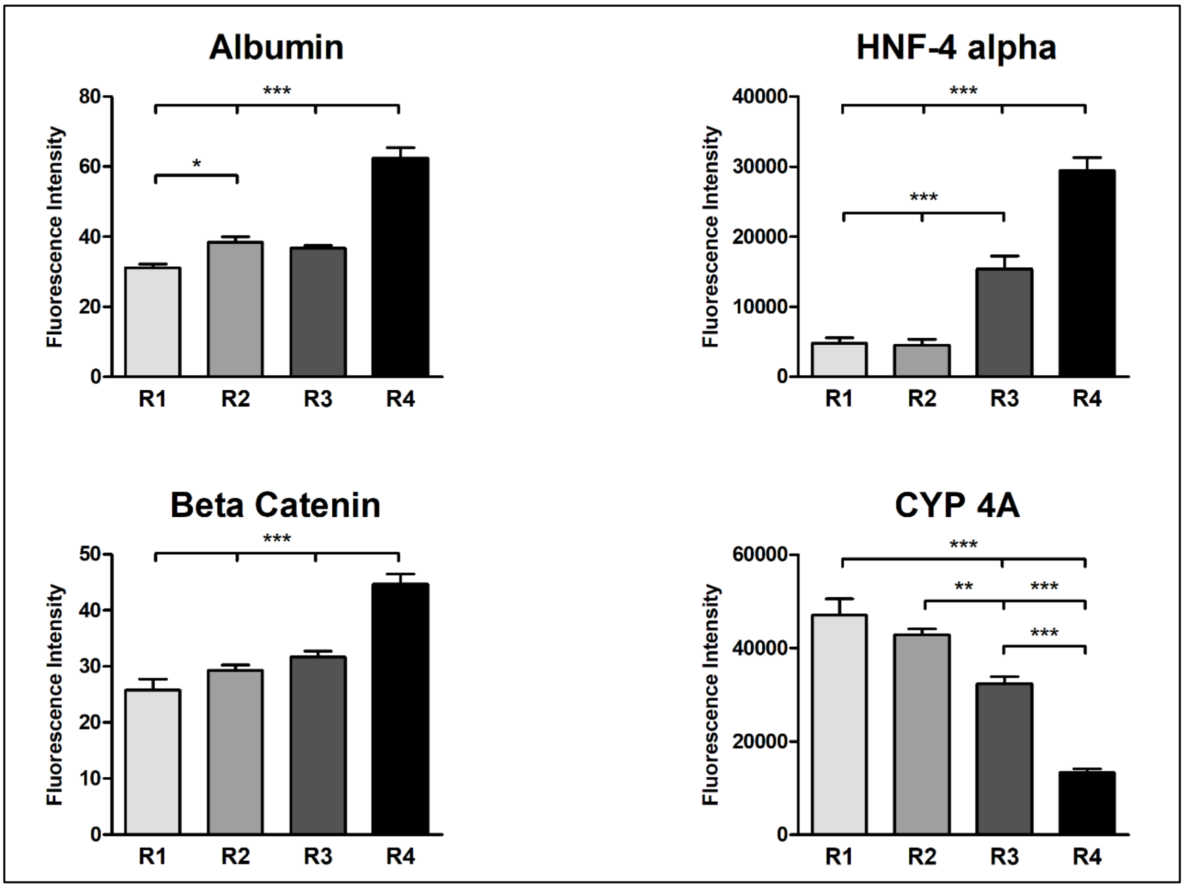

MATERIALS AND METHODS

Cell monolayer processing in Transmission Electron Microscopy (TEM)

Differentiated hAEC monolayers cultured on Geltrex were fixed with 2.5% glutaraldehyde (Electron Microscopy Science, Hatfield, PA 19440, USA) in 0.1 M cacodylate buffer (Electron Microscopy Science), pH 7.2-7.4, for 1 h at room temperature. They were then washed in 0.1 M cacodylate buffer and post-fixed in 1% osmium tetroxide for 1 h at 4°C. After being dehydrated in progressively higher concentrations of alcohol, cell monolayers were embedded in Spurr resin (Electron Microscopy Science) and cut with a Reichert ultramicrotome (Reichert, Inc., Teramo, Italy). Ultrathin sections were mounted on nickel grids, stained with uranyl acetate and lead citrate, and observed with a ZEISS 109 transmission electron microscope equipped with a Gatan-Orius 830Z00W44 camera and Digital Micrograph application (Gatan GmbH, Ingolstadterstr. 12, D-80807 München, Germany) for acquiring, visualizing, analysing, and processing digital image data.

RNA Isolation and qRT-PCR

Total RNA was isolated using TRIzol reagent (Thermo Fisher Scientific) according to the manufacturer's protocol. RNA integrity and purity were confirmed with 1% agarose gel electrophoresis and OD260/OD280 nm absorption ratio >1.8. Two grams of DNase-I-treated RNA of each sample were reverse-transcribed with PCR using Promega reagents (Madison, WI, USA). The resulting complementary DNA (cDNA) was analysed with real-time q-PCR using specific TaqMan assays and TaqMan Gene Expression Master Mix on a StepOne System (all from Thermo Fisher Scientific). Human-specific assays are listed in Supporting Table 1. For all assays, the thermal profile was as follows: 50°C for 2 minutes, 95°C for 10 minutes, 45 cycles at 95°C for 15 seconds, and 60°C for 1 minute. Fold change was calculated with the $2^{-\Delta\Delta CT}$ method [1] .

Immunofluorescence

At the end of the differentiation protocol, hAEC were fixed in 3% formalin for 10 min, washed twice with 1X PBS and permeabilized with 0.1% Triton X-100 for 10 min. After blocking in Goat Serum for 30 min, cells were incubated with primary antibodies (Supporting Table 1) for 2 h. Cells were then washed in 1X PBS, incubated with the secondary antibody (Supporting Table 1) for 1 h (except for Albumin, which was a FITC-conjugated antibody) and washed in 1X PBS. Cells were counterstained with DAPI (Abcam, Cambridge, UK) for 10 min and observed under UV light with a IX71 fluorescence microscope (Olympus, Tokyo, Japan).

Image analysis, graphical representation of results and statistical analysis

Acquired microscopic images were processed for quantitative analysis using Image-Pro Premier software (Media Cybernetics, Rockville, MD, USA). Graphical representation of quantitative results and statistical analysis were performed using Prism 5 software (GraphPad Software, La Jolla, CA, USA). Data were plotted as mean ± SE.

REFERENCES

[1] **Schmittgen TD, Livak KJ**. Analyzing real-time PCR data by the comparative C_T method. *Nat. Protoc.* 2008; 3; 1101–8.

Supporting Table 1. List of Reagents

**qRT-PCR Target Genes**	**Taqman Assay ID** (Thermo Fisher Scientific)	
β2 microglobulin	Hs99999907_m1	
Albumin	Hs00910225_m1	
Hepatocyte nuclear factor 4α	Hs00230853_m1	
α1 antitrypsin	Hs01097800_m1	
Cytochrome P450 3A7	Hs00426361_m1	
Cytochrome P450 3A4	Hs00430021_m1	
UDP glucuronosyltransferase 1A1	Hs02511055_s1	
**Primary Antibodies**		
Albumin - FITC conjugated	Abcam ab53435	Dilution 1:50
β-Catenin	Abcam ab6302	Dilution 1:100
HNF4α	Abcam ab92378	Dilution 1:100
CYP 4A	Abcam ab3573	Dilution 1:100
**Secondary Antibody**		
Anti-rabbit – Dylight 488 conj.	Abcam ab96899	Dilution 1:250

Supporting Table 2. Comparison of gene expression levels relative to β2 microglobulin.

qRT-PCR Target Genes		Undifferentiated hAEC (n=5)	hAEC-Hep (n=5)				Fetal Human Hepatocytes (n=5)	Adult Human Hepatocytes (n=8)
			R1	R2	R3	R4		
Albumin	mean ± SD	0.000060 ±0.000056	0,00030 ±0,00011	0,00078 ±0,00022	0,00096 ±0,00009	0,00112 ±0,00024	40.20 ±13.23	30,89 ±16,34
	% Undif. hAEC		506%	1293%	1597%	1867%		
	% Fetal HH		0,0008%	0,0019%	0,0024%	0,0028%		
	% Adult HH		0,0010%	0,0025%	0,0031%	0,0036%		
CYP 3A7	mean ± SD	0,0000080 ±0,0000080	0.00211 ±0.00023	0.00016 ±0.00013	0.00020 ±0.00004	0.00024 ±0.00011	0,15 ±0,05	0,016 ±0,022
	% Undif. hAEC		26389%	2010%	2544%	2941%		
	% Fetal HH		1,39%	0,11%	0,13%	0,16%		
	% Adult HH		13,44%	1,02%	1,30%	1,50%		
CYP3A4	mean ± SD	0,0000090 ±0,0000040	0.000076 ±0.000020	0.000029 ±0.000036	0.000017 ±0.000015	0.000016 ±0.000019	0,0041 ±0,0014	7,20 ±4,83
	% Undif. hAEC		848%	328%	188%	178%		
	% Fetal HH		1,87%	0,72%	0,42%	0,39%		
	% Adult HH		0,00106%	0,00041%	0,00023%	0,00022%		
HNF4α	mean ± SD	0,000000025 ±0,000000049	0.00000177 ±0.00000354	0.00000001 ±0.00000002	0.00000538 ±0.00000682	0.000001059 ±0.00000977	0,013 ±0,006	0,045 ±0,017
	% Undif. hAEC		7210%	51%	21944%	43166%		
	% Fetal HH		0,013%	0,000%	0,040%	0,079%		
	% Adult HH		0,0039%	0,0000%	0,0119%	0,0235%		
A1AT	mean ± SD	0,00065 ±0,00078	0.0029 ±0.0009	0.0028 ±0.0016	0.0011 ±0.0005	0.0017 ±0.0015	1,95 ±0,55	7,62 ±4,27
	% Undif. hAEC		453%	427%	173%	271%		
	% Fetal HH		0,15%	0,14%	0,06%	0,09%		
	% Adult HH		0,038%	0,036%	0,015%	0,023%		
UGT1A1	mean ± SD	0,00012 ±0,00009	0.00040 ±0.00037	0.00123 ±0.00082	0.00131 ±0.00041	0.00137 ±0.00088	0,000007 ±0,000006	0,093 ±0,042
	% Undif. hAEC		339%	1044%	1107%	1156%		
	% Fetal HH		5744%	17668%	18739%	19564%		
	% Adult HH		0,43%	1,32%	1,40%	1,47%		
